# DNA Origami Vesicle Sensors with Triggered Single‐Molecule Cargo Transfer

**DOI:** 10.1002/anie.202408295

**Published:** 2024-10-30

**Authors:** Ece Büber, Renukka Yaadav, Tim Schröder, Henri G. Franquelim, Philip Tinnefeld

**Affiliations:** ^1^ Department of Chemistry Center for NanoScience Ludwig-Maximilians-University Butenandtstraße 5–13 81377 Munich Germany; ^2^ Interfaculty Centre for Bioactive Matter Leipzig University Deutscher Platz 5 (BBZ) 04103 Leipzig Germany

**Keywords:** Affinity interactions, DNA origami, lipid vesicles, cargo transfer, single-molecule FRET

## Abstract

Interacting with living systems typically involves the ability to address lipid membranes of cellular systems. The first step of interaction of a nanorobot with a cell will thus be the detection of binding to a lipid membrane. Utilizing DNA origami, we engineered a biosensor with single‐molecule Fluorescence Resonance Energy Transfer (smFRET) as transduction mechanism for precise lipid vesicle detection and cargo delivery. The system hinges on a hydrophobic ATTO647N modified single‐stranded DNA (ssDNA) leash, protruding from a DNA origami nanostructure. In a vesicle‐free environment, the ssDNA coils, ensuring high FRET efficiency. Upon vesicle binding to cholesterol anchors on the DNA origami, hydrophobic ATTO647N induces the ssDNA to stretch towards the lipid bilayer, reducing FRET efficiency. As the next step, the sensing strand serves as molecular cargo that can be transferred to the vesicle through a triggered strand displacement reaction. Depending on the number of cholesterols on the displacer strands, we either induce a diffusive release of the fluorescent load towards neighboring vesicles or a stoichiometric release of a single cargo‐unit to the vesicle on the nanosensor. Ultimately, our multi‐functional liposome interaction and detection platform opens up pathways for innovative biosensing applications and stoichiometric loading of vesicles with single‐molecule control.

## Graphical abstract

This study presents a DNA origami‐based biosensor for precise lipid vesicle detection and cargo delivery using single‐molecule Fluorescence Resonance Energy Transfer (smFRET). The hydrophobic ATTO647N‐modified single‐stranded DNA (ssDNA) leash on the sensor detects vesicle binding via FRET changes and delivers molecular cargo through triggered strand displacement reaction. This platform offers innovative applications in biosensing and controllable stoichiometric loading of vesicles.

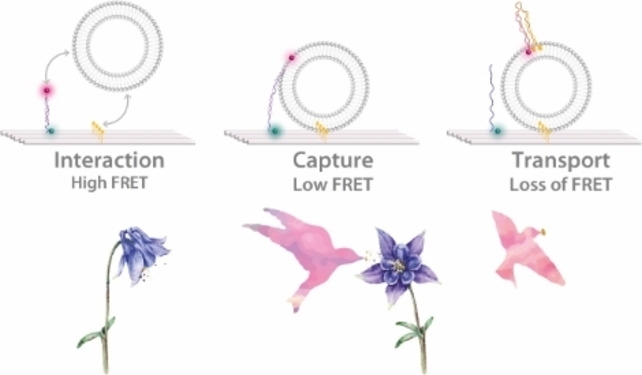

## Introduction

In the rapidly advancing field of nanotechnology, the development of dynamic systems that respond to specific molecular signals is becoming increasingly important. These systems, capable of translating molecular behavior into practical applications, have the potential to reshape areas such as biosensing, targeted therapeutics, and precise engineering at the nanoscale. Central to these advancements is the DNA origami technique,[^1^, ^2^, ^3^, ^4^, ^5^, ^6^, ^7^] which offers a reliable and customizable framework for designing nanoscale interactions by having stoichiometric and positional control over the DNA structure and attached functional elements. DNA origami, utilizing the innate programmability of DNA sequences, enables the design and realization of intricate nanostructures with exceptional precision. This unique capability has fostered innovations across nanotechnology, particularly in biosensing.[^8^, ^9^, ^10^, ^11^] With the ability to design custom sensors tailored for specific molecular targets, DNA origami emerges as a powerful tool to address the challenges posed by complex biological systems.[^12^, ^13^, ^14^, ^15^, ^16^, ^17^, ^18^, ^19^]

Among the challenges, membrane systems and especially, lipid vesicles stand out. These membranous sacs play pivotal roles in diverse cellular functions, from molecular transfer and signaling to compartmentalization.[^20^, ^21^] Therefore, detecting and characterizing lipid vesicles is of great importance. Sensors based on DNA origami can offer a subtle understanding of how lipid vesicles behave, with the potential to probe, detect, and even manipulate their activities.[^22^, ^23^, ^24^, ^25^, ^26^, ^27^] Bridging the innovative capabilities of DNA origami to the intricate world of lipid vesicles can provide deeper insights into vesicular behaviors and potentially unlock new therapeutic opportunities.[^28^, ^29^, ^30^, ^31^]

DNA origami has uses beyond just detection. As modern medicine and technology advance, the need for precise and controlled cargo transportation at the nanoscale becomes increasingly evident. From targeted drug delivery to the transfer of specific molecular agents, the ability to move and release cargo with specificity could reshape therapeutic strategies. In one of the pioneering works, Douglas et al. developed a DNA nanorobot that delivers payloads to cells and changes its structure to release them, showing promise for targeted cell therapy.^[32]^ Thubagere et al. created a self‐powered DNA robot with three functional domains that can move across a DNA origami sheet and sort two types of molecular cargoes to their respective destinations using a simple algorithm.^[33]^ In a more recent work involving lipid vesicles, Baumann et al. created a DNA mesh around lipid vesicles for drug delivery, releasing the dye calcein upon triggering. This method increased cytotoxicity in HEK293T cells and holds potential for targeted chemotherapy delivery.^[34]^ In our research, we have developed a multifunctional system that is not only capable of sensing the presence of a vesicle, but also has the potential to deliver a desired cargo directly to the vesicle it is attached to. By leveraging the capabilities of DNA origami, integrating the precision of single‐molecule FRET (smFRET), and utilizing specific binding interactions, we have created a biosensing and single‐molecule cargo transfer system.

Building on our experience with DNA origami nanosensors for lipid vesicle characterization,^[27]^ herein we introduce a DNA origami biosensor tailored for lipid vesicle detection. This system utilizes an ATTO647N labeled single‐stranded DNA (ssDNA) protrusion, a donor dye ATTO542, and cholesterol anchors. It capitalizes on smFRET and the affinity interactions of ATTO647N, allowing for real‐time vesicle sensing and the potential for triggered cargo transport of single molecules. In our system, ATTO647N serves a dual role: not only it is the acceptor dye in the FRET process, but its hydrophobic nature also drives its interaction with lipid bilayers. It sets the stage for innovative biosensing applications and the transport of molecules, particularly by delivering desired single cargoes to lipid membranes in a localized or diffusive manner.

## Results and Discussion

**Figure 1:**
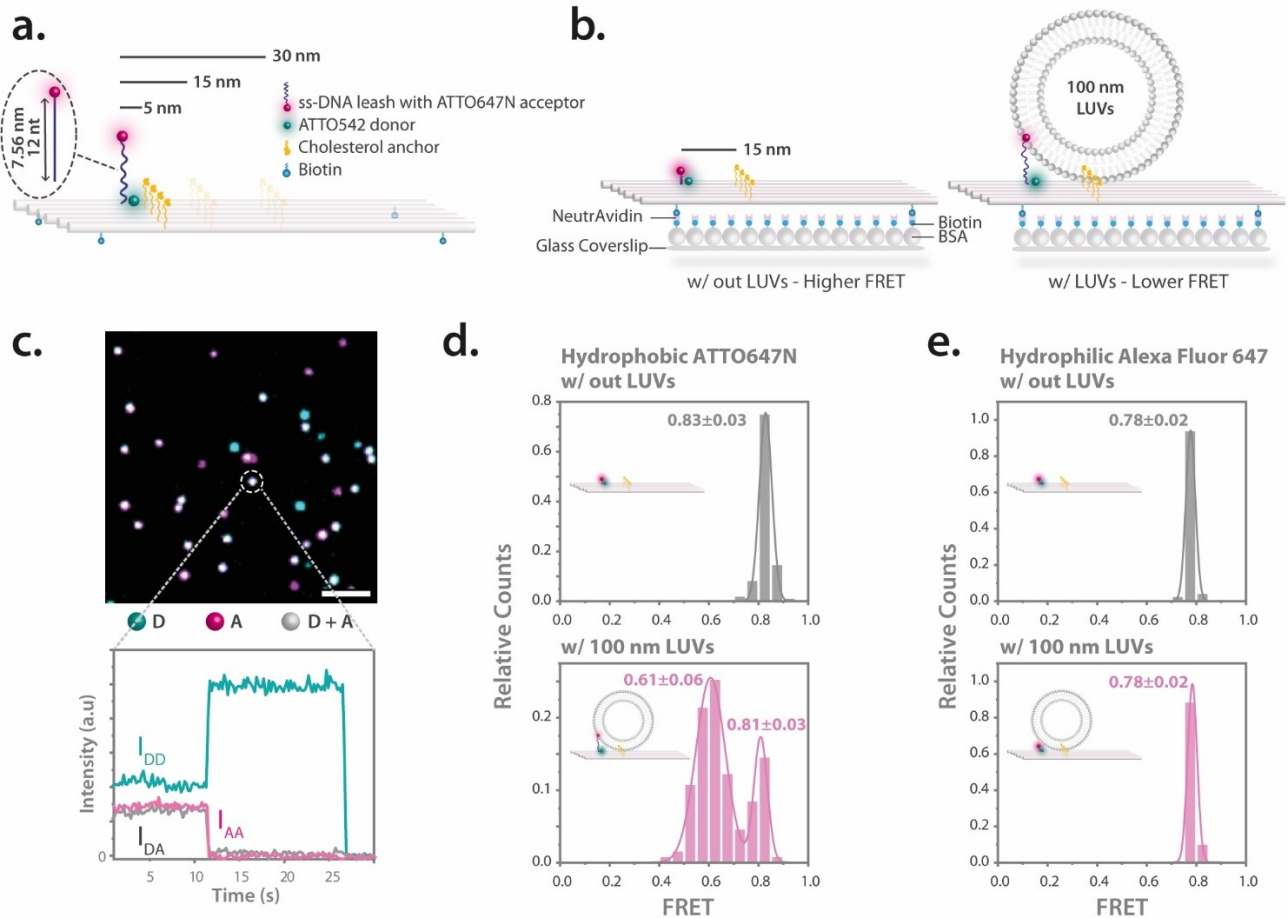
Concept and validation of the vesicle sensor. (a) A rectangular DNA origami base equipped with a sensing unit consisting of a 12 nt ssDNA leash labeled with an ATTO647N acceptor and an ATTO542 donor positioned on the DNA origami base. The extended length of the ssDNA leash is 7.56 nm. Cholesterol anchors, strategically placed at various distances for vesicle capture, and biotin moieties for surface attachment are also featured. Note: Illustrations are not to scale. Please refer to Supplementary Figure S2 for a scaled diagram. (b) These sensors have the capability to bind to BSA‐Biotin‐NeutrAvidin‐treated glass coverslips using biotin moieties. Without lipid vesicles (left), the ssDNA sensing probe adopts a coiled configuration, positioning it closer to the donor dye, resulting in higher FRET. When lipid vesicles are present (right), the sensing probe elongates to permeate the lipid bilayer, driven by the hydrophobic nature of the ATTO647N fluorophore. Due to the increased distance between the probe and the donor dye, a decreased FRET is observed. (c) The image at the top presents a superimposed TIRF image with the donor dye (D) shown in cyan, and the acceptor (A) in magenta. Gray spots denote sensors that incorporate both the donor and acceptor dyes. An exemplary single‐molecule FRET (smFRET) trace at the bottom illustrates the fluorescence intensity over a period, detailing the donor excitation–donor emission (I_DD_) channel (cyan), the donor excitation–acceptor emission (I_DA_) channel (gray), and the acceptor excitation–acceptor emission (I_AA_) channel (magenta). Mean FRET efficiencies are calculated from the I_DD_ and I_DA_ channels. Scale bar is 5 μm. (d) FRET efficiency distributions for vesicle sensors with cholesterol anchors placed at 15 nm distance from the probe position, in scenarios both without (top) and with (bottom) lipid vesicles. Distributions are shown for the hydrophobic sensing probe with ATTO647N and (e) the hydrophilic control probe with Alexa Fluor 647. Accompanying illustrations in the plots suggest potential conformations of the probe. The error refers to the standard deviation (SD). Number of molecules used in the FRET calculations are as follows; for the sensor with hydrophobic ATTO647N probe 111 molecules were used without LUVs and 131 with LUVs and for the sensor with hydrophilic Alexa Fluor 647 probe 127 molecules were used without LUVs and 112 with LUVs.

The vesicle sensor is crafted from a rectangular DNA origami nanostructure[^2^, ^35^] with the dimensions of 70×100 nm. It features a 12 nucleotide (nt) long ssDNA leash, modified with an ATTO647N fluorophore which serves as the main probe for vesicle sensing (Figure 1a). This probe, due to its hydrophobic and cationic properties, anchors itself in phospholipid vesicles, a behavior noted in prior studies.[^36^, ^37^, ^38^] Moreover, the sensor is equipped with an internally labeled ATTO542 fluorophore, serving as a donor for FRET (Figure 1a and Figure S1). We postulate that the relative position of the acceptor probe will change based on the presence or absence of lipid vesicles (Figure 1b). In a vesicle‐free environment, the ssDNA probe naturally adopts a coiled conformation due to its flexible and dynamic nature. This coiling behavior is driven by the entropy of the polymer, hydrophobic interactions of the bases, and electrostatic repulsion along the phosphate backbone. Upon vesicle binding to cholesterol anchors on the DNA origami, the interaction of the hydrophobic ATTO647N dye with the membrane stretches the ssDNA out towards the lipid bilayer, reducing FRET efficiency due to the increased distance between the donor and acceptor dyes. For anchoring the sensor to modified glass coverslips, biotin attachments are located at the four corners of the DNA origami structure (Figure 1a and Figure S1). The sensor includes four cholesterol‐based anchors (Figure 1a and Figure S1) to facilitate the capture of lipid vesicles by providing multiple interaction sites. The parallel arrangement of these anchors ensures structural stability and maintains a defined distance between the sensor unit and the cholesterol line.[^39^, ^40^]

To visualize individual vesicle sensors, we employed smFRET using total internal reflection fluorescence microscopy (TIRF) on a commercial fluorescence microscope (Nanoimager S, ONI Ltd., UK) with green‐red alternating laser excitation (ALEX).[^41^, ^42^] Intensity transients of single spots were extracted from TIRF videos using the iSMS software based on Matlab.^[43]^ In Figure 1c, a false‐color image displays donor dye emission in cyan, acceptor in magenta, and their overlay in gray. We verified the fluorescence as originating from single vesicle sensors by observing single‐step photobleaching. For instance, in Figure 1c, the acceptor dye photobleaches after 11 seconds, leading to an increased, unquenched donor fluorescence (I_DD_), while the FRET signal (I_DA_) drops to zero. This synchronized response confirms the presence of a single DNA origami structure exhibiting FRET. We further tracked acceptor emission following its excitation (I_AA_) to study associated photophysical behaviors and prioritize initial acceptor bleaching events. From the intensity data of both the I_DD_ and I_DA_ channels during energy transfer, the FRET efficiency of individual sensors was quantified as

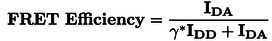




Here, I_DA_ is corrected to account for the direct excitation of the acceptor at the donor excitation wavelength and for any donor emission leakage into the acceptor emission channel. The γ correction factor compensates for the different quantum yields of the dyes and wavelength‐dependent efficiencies of the detection (see Supporting Information for detailed materials and methods). For each sample, we analyzed around 100 molecules and represented the FRET efficiencies in histograms, complemented with Gaussian fits where relevant.

To validate our design, we contrasted the FRET efficiencies of the sensor both in the absence and presence of lipid vesicles. For this experiment, considering the highest possible overlap with the leash length and 100 nm lipid vesicles, we used sensors with cholesterol anchors placed at a 15 nm distance from the probe position. The calculated optimal distance using the ideal chain model for a 12 nt ssDNA leash and 50 nm radius vesicles is approximately 14.93 nm, which closely matches our experimental setup (Please refer to the Supporting Information Appendix I for the calculation details). After immobilizing the sensors on BSA‐Biotin‐NeutrAvidin‐treated glass coverslips, smFRET measurements were performed. In the absence of vesicles, the mean FRET value was 0.83±0.03 (standard deviation of the mean, SD). Upon introducing 1 nM of 100 nm 1,2‐Dioleoyl‐sn‐glycero‐3‐phosphocholine (DOPC) vesicles (see Supporting Information for the details of lipid vesicle preparation) with a 1‐hour incubation, we observed an additional population with a mean FRET value of 0.61±0.06 (Figure 1d). This shift confirms the effective binding of lipid vesicles to the DNA origami platform and suggests the binding of the sensing unit to the vesicle membrane. A remaining fraction of 20 % with a mean FRET of 0.81±0.03 likely represents either sensors that do not have vesicles in their vicinity or sensors where the probe is unable to successfully engage with the incorporated vesicles.

To further substantiate the role of affinity interactions in the sensor mechanism, the hydrophobic ATTO647N fluorophore was substituted with Alexa Fluor 647 on the ssDNA probe. Previous research has demonstrated that Alexa Fluor 647 exhibits minimal interaction with lipid vesicles due to its hydrophilic nature.[^36^, ^44^] When analogous experiments were conducted without and with lipid vesicles for the sensors equipped with Alexa Fluor 647, the mean FRET values remained consistent at 0.78±0.02 (Figure 1e). The notably homogeneous and narrow distributions for the Alexa Fluor 647 probe further underscore the assertion that the distinct behavior of the sensor arises from the hydrophobic affinity interactions between the ATTO647N probe and lipid vesicles.

Building on our observations, we investigated the influence of the distance of the FRET probe to the cholesterol anchors. In addition to sensors with cholesterol anchors at 15 nm distance, we assembled vesicle sensors with cholesterol moieties positioned at 5 nm and 30 nm distance from the probe. Each sensor was subjected to smFRET studies both with and without lipid vesicles.

**Figure 2:**
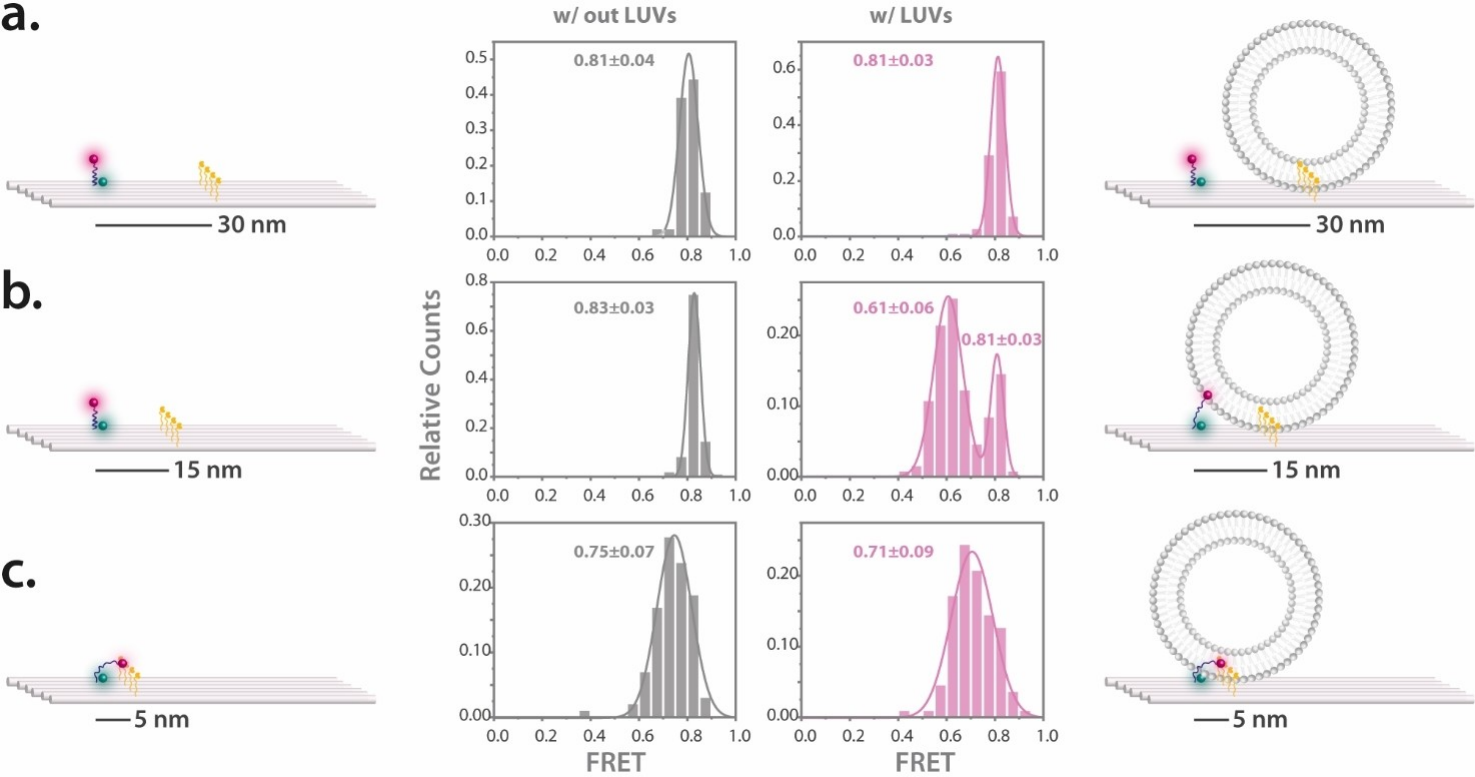
Influence of the cholesterol anchoring distances on the sensor both in the absence and presence of lipid vesicles. The distributions of FRET efficiency for the vesicle sensors, both without and with lipid vesicles, is depicted for (a) 30 nm, (b) 15 nm, and (c) 5 nm cholesterol‐FRET probe distances. Illustrations accompanying the data highlight the positions of the cholesterol anchors and potential movement patterns of the sensing probe. The error refers to the standard deviation (SD). Number of molecules used in the FRET calculations are as follows; for the sensor with 30 nm cholesterol anchors 97 molecules were used without LUVs and 113 with LUVs, for the sensor with 15 nm cholesterol anchors 111 molecules were used without LUVs and 131 with LUVs, and for the sensor with 5 nm cholesterol anchors 85 molecules were used without LUVs and 112 with LUVs.

For the sensor with the cholesterol anchors at a 30 nm distance from the leash, the smFRET results (Figure 2a) reported mean FRET values of 0.81±0.04 and 0.81±0.03 for scenarios without and with vesicles, respectively. The data suggests that, even when vesicles are present, the probe remains in its coiled state because the vesicles are too distant to be reached by the ATTO647N anchor. This design confirms that the FRET contrast arises solely when the ATTO647N probe anchors into the lipid bilayer—an event only possible when the vesicle is close enough to allow the probe to stretch toward it.

In the sensor variant with cholesterols at a 15 nm distance from the sensor, as already presented in Figure 1d, a clear FRET shift occurred based on vesicle presence, underlining the effect of proximity (Figure 2b). However, the results took a captivating turn when cholesterol anchors were only 5 nm from the probe. Independent of lipid vesicle presence, these sensors exhibited wider FRET distributions with comparable mean values (0.75±0.07 without vesicles and 0.71±0.09 with vesicles, Figure 2c), yet distinct from the other tested distances. We attribute this observation to the strong hydrophobic interactions that allow the ATTO647N probe to already interact with closely positioned cholesterol anchors. Consequently, given the distinctive and reliable FRET shift observed with the 15 nm spacing, we chose to utilize sensors with cholesterol moieties at this distance for the remainder of our study. Additionally, a noteworthy observation was that, upon testing the 5 nm cholesterol configuration with a control system featuring Alexa Fluor 647, there was no apparent difference in FRET distributions (Figure S3).

In our pursuit to understand the sensor‘s response to varying lipid vesicle sizes, we further examined its behavior with 50 nm, and 200 nm DOPC vesicles using the 15 nm cholesterol as well as a 20 nm cholesterol configuration (Figure S4‐a). Notably, while the 15 nm cholesterol‐based sensor exhibited minimal variation across vesicle sizes (Figure S4), the 20 nm variant revealed a discernible peak in its interaction with the 200 nm DOPC vesicles, as illustrated in Figure S4‐c. This new population, however, constituted less than 10 % of the overall sample. These findings indicate that the positioning of cholesterol in relation to the probe can influence the affinity of the sensor towards differently sized vesicles. Although the observed new distribution is a small fraction of the overall sample, it underscores the importance of meticulous design and adaptation in probe creation for vesicle interactions. Tailoring probes to align with specific vesicle dimensions can optimize their performance and extend their versatility in multiple disciplines.[^45^, ^46^, ^47^, ^48^]

**Figure 3:**
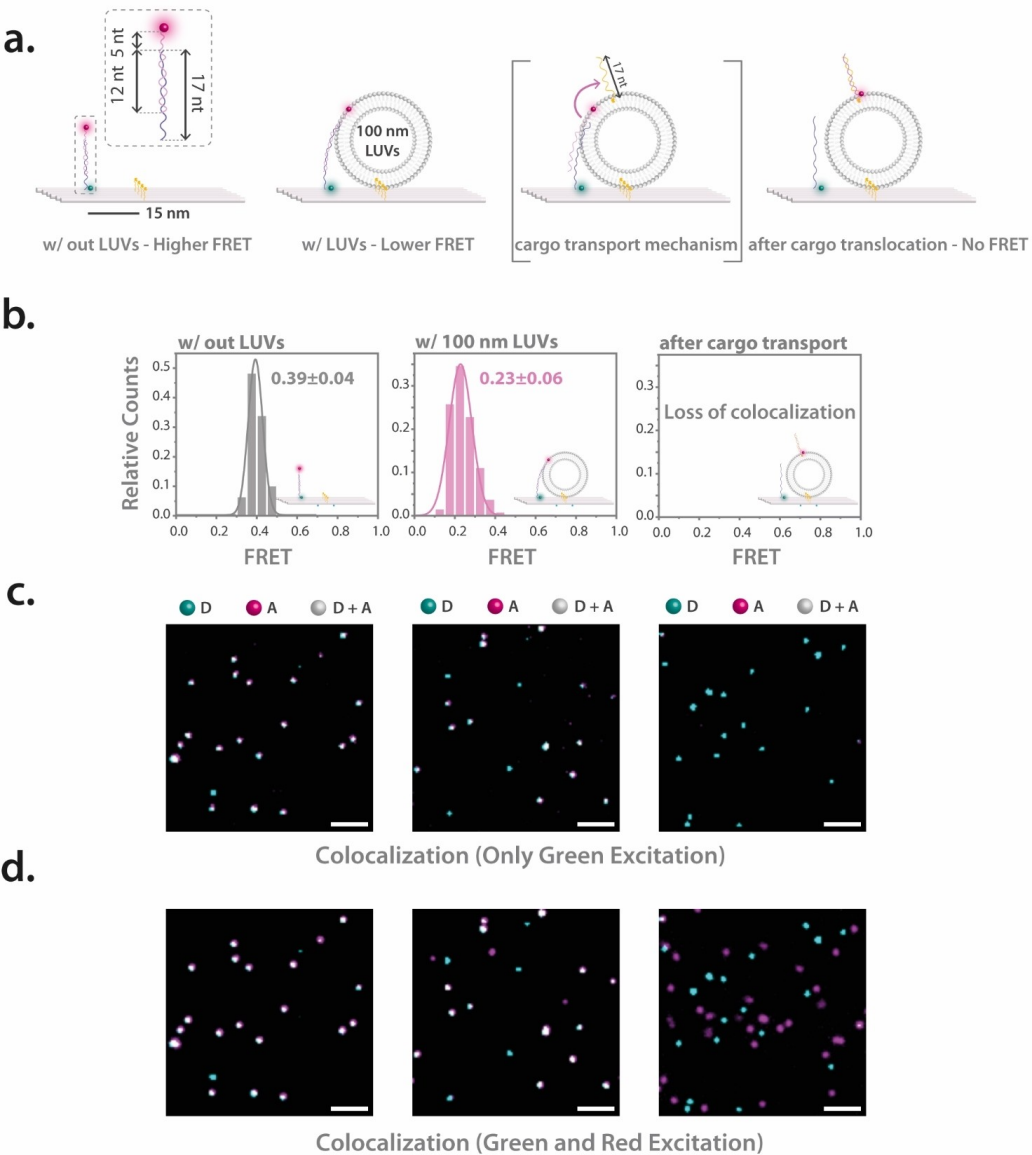
Triggered cargo transfer to the lipid vesicles. (a) Schematic illustrations detailing the cargo transfer system. A 17 nt strand protrudes from the DNA origami base, binding to a complementary 17 nt strand labeled with ATTO647N, leaving a 5 nt toehold exposed. The system initially exhibits higher FRET levels in the absence of lipid vesicles. Upon interaction with lipid vesicles, the ATTO647N‐labeled strand anchors into the lipid bilayer, leading to reduced FRET. After cargo transfer by strand displacement, the system shows no FRET due to increased separation between the FRET pair. (b) FRET histograms displaying the FRET characteristics of the system under different conditions: without lipid vesicles (left), with lipid vesicles (middle), and post‐cargo transfer (right), where no FRET is observed. (c) Superimposed TIRF images after green‐only excitation. The systems without vesicles (left) and with vesicles (middle) show clear FRET, evidenced by colocalization. After cargo transfer (right), only green spots are observed, indicating the loss of FRET and absence of colocalization. (d) Superimposed TIRF images after both green and red excitation. In the absence (left) and presence (middle) of lipid vesicles, high degree of colocalization is observed. Following cargo transfer (right), the colocalization is lost, coinciding with the absence of FRET. However, numerous red spots remain at other locations. Scale bar is 5 μm. Number of molecules used in the FRET calculations are as follows; 160 molecules were used without LUVs and 136 with LUVs.

Building upon the insights from our prior experiments and recognizing the transformative potential of cargo transport systems in molecular and nanoscale research, we delved deeper into an intricate investigation employing the ATTO647N FRET acceptor probe as membrane cargo within a strand displacement system. A 17 nt ssDNA was protruding from the probe position on the DNA origami and a 17 nt ATTO647N probe was attached to this protrusion, preserving a 5 nt toehold at the forefront (Figure 3a). The cargo transfer system involved a 17 nt cholesterol‐modified displacer strand, which has a stronger affinity for the ATTO647N probe. Upon interaction, this displacer strand binds to the probe at the toehold segment, leading to its displacement from the origami structure. More precisely, as we noted higher FRET values without vesicles and a decrease upon vesicle introduction, we further anticipated witnessing a loss of the FRET signal but a persisting colocalization of green and red emission after the displacement of the ATTO647N probe. We initially expected colocalized spots after introducing the displacer strand because the ATTO647N‐labeled cargo, upon displacement, was designed to remain associated with the lipid vesicles bound to the DNA origami structure. This expectation is based on the assumption that the cholesterol‐modified displacer strand would transfer the cargo directly to the vesicles captured by the DNA origami, maintaining proximity due to the membrane interaction. Initial smFRET imaging without lipid vesicles revealed a mean FRET value of 0.39±0.04. This lower initial FRET value compared to the previous sensing system (0.83±0.03) is attributed to the structural differences between the two designs. In the initial vesicle sensing system, the probe is a flexible ssDNA leash, allowing close proximity of the donor and acceptor fluorophores. In contrast, the cargo transport system has a longer (17 nt) protrusion including a 12 nt duplex DNA region, resulting in a more extended configuration for the duplex part and greater distance between the donor and acceptor, thereby reducing the FRET efficiency. Presence of lipid vesicles reduced this value to 0.23±0.06, corroborating the consistent working principle of the sensor (Figure 3b and Figure 3c, left and middle panels). This new vesicle sensing and cargo transporting system design showed a unimodal distribution after LUV incorporation, indicating no unbound fraction (Figure 3b). By estimating the bound fractions, we observed a notable increase from 80 % in the initial sensor system to 95 % in the cargo transfer system design (Figure S5). It is noteworthy that both ssDNA and dsDNA probes exhibit marked FRET changes upon vesicle docking. While ssDNA is flexible and can adopt various conformations, dsDNA is more rigid due to its stable double‐helix structure.[^49^, ^50^, ^51^, ^52^] In our design, the cargo transfer system design includes a probe having both dsDNA and ssDNA regions. The flexibility of the ssDNA parts allows for significant conformational changes, even when combined with the more rigid dsDNA segments.

The experiment took an interesting development when 1 nM of the 17 nt cholesterol‐labeled displacer strand was introduced. The FRET signal on the DNA origami nanosensors immediately vanished, yet a non‐colocalizing red emission, primarily at neighboring spots, persisted on the images (Figure 3b and Figure 3c, right panel). This red emission provided compelling evidence of the probe being successfully detached from the origami. Prior to cargo transfer, the system exhibited a high degree of colocalization, as evidenced in Figure 3d, left and middle panels. However, upon introduction of the displacer strand, instead of the anticipated colocalized spots, numerous red‐only spots appeared (Figure 3d, right panel). As single cholesterol can diffuse in and out of lipid vesicles,[^53^, ^54^] the transferred probe can subsequently relocate to surrounding lipid vesicles. We theorize that the DNA displacer strand and cargo with a single cholesterol still exhibits mobility across vesicles and can bind to free lipid vesicles also adhering to the surface.^[53]^ This phenomenon resulted in diminished colocalization of the red fluorescent cargo within the DNA origami sensors (and their trapped vesicles). Furthermore, the evident post‐displacement motion in these non‐colocalized red spots hints at the dynamic diffusion of the fluorescent ATTO647N cargo transfered with single‐cholesterol‐modified displacer strand in and across lipid vesicles (Supporting Information Movie 1). Supporting these observations, when lipid vesicles in the system were deliberately ruptured using a 0.05 % Tween20 buffer,^[55]^ almost all red signals disappeared, reaffirming the specific vesicle‐probe interaction (Figure S6‐d). A separate evaluation of the impact of Tween20 on the system showed it only disrupts lipid vesicles and thereby resets the sensor to its original state without vesicles (see Figure S7). To fortify our hypothesis, a control test without vesicles was carried out. Upon introducing the cholesterol‐labeled displacer strand, a near‐complete disappearance of the red signal was evident which highlights the effectiveness of the strand in displacing the probe (Figure S8). In the absence of lipid vesicles, the displacement process was considerably slower, taking almost an hour for the red signal to fade, whereas in the presence of the vesicles, the red signal faded in just tens of seconds. Supposedly, the cholesterol displacer strand first binds to the lipid vesicles, locally upconcentrating the displacer strand. This upconcentration close to the probe results in increased transfer kinetics.

**Figure 4:**
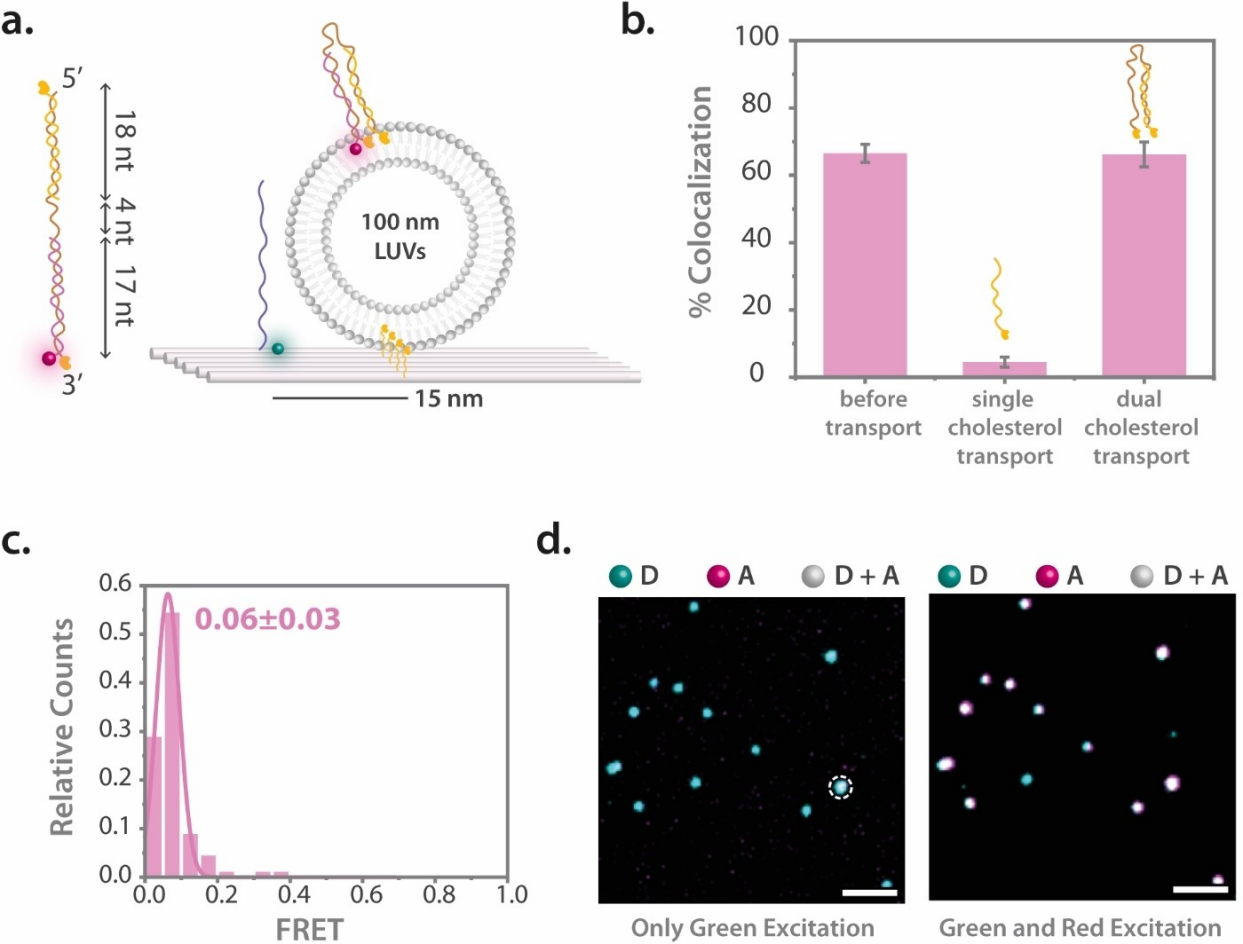
Targeted cargo transfer using a dual cholesterol‐labeled displacer. (a) Illustration of the dual‐cholesterol displacer consisting of a 39 nt ssDNA with a 3′ end cholesterol‐TEG modification, linked to an ATTO647N‐labeled 17 nt ssDNA at its 3′ end. Additionally, an 18 nt ssDNA with a 3′ end cholesterol modification is bound to the 5′ end of the long strand, separated by a 4 nt thymine (T) spacer. On the right is the depiction of DNA origami vesicle sensor with a bound lipid vesicle, illustrating the post‐transfer scenario with the new construct. (b) % colocalization of the cargo transfer systems with single and dual cholesterol displacers compared to the system prior cargo transfer. (c) Histogram of FRET efficiency post‐cargo transfer, displaying negligible FRET values, indicative of successful cargo displacement with minimal proximity between FRET pairs. (d) Superimposed TIRF microscopy images following cargo transfer; left image shows detection after only green excitation to highlight the loss of FRET with an encircled spot to exemplify a rare FRET event, and the right image under green and red excitation, illustrating the colocalization and confirming the presence of cargo within the system. Scale bar is 5 μm. Number of molecules used in the FRET calculations is 90.

After our initial observations of cargo transfer using single‐cholesterol modified displacer strands, we aimed to refine the specificity of cargo transfer to ensure that it remains within the vesicle bound to the DNA origami structure, instead of spontaneously translocating to neighboring vesicles. As indicated before, previous research has shown the mobility of cholesterol‐labeled DNA strands across vesicles.[^53^, ^54^] To address this, we adopted a strategy involving the use of a dual cholesterol‐labeled DNA strand for the strand displacement reaction. We engineered a 39 nt long ssDNA with a cholesterol‐TEG modification to which our ATTO647N‐labeled cargo strand can bind at the 3’ end. An additional 18 nt ssDNA with a 3′ end cholesterol‐TEG modification was designed to bind to the 5′ end of the long DNA strand creating a double‐stranded configuration with enhanced vesicle binding stability. With a 4 nt Thymine (T) spacer between the hybridized regions, we aimed to provide flexibility to the construct (Figure 4a).

Prior to experimentation, the two cholesterol‐modified strands were pre‐incubated in a 1 : 1 molar ratio at 37 °C for 2 hours, forming the new displacer strand. This construct was introduced to the system following the standard protocol for vesicle incubation and FRET monitoring. Upon addition of 1 nM of the dual cholesterol‐labeled displacer strand, we observed an immediate loss of FRET but a remaining colocalization (Figure 4b). The loss of FRET was supported with a histogram showing negligible FRET values (Figure 4c). The occasional FRET events (encircled spot in the left image in Figure 4d) are likely due to sensors either failing to capture vesicles or to cargo translocated near the FRET‐sensitive region of the sensor. Contrary to earlier findings, in this case, we observed a high level of control over the cargo transfer, as evidenced by the complete loss of FRET population (Figure 4c). This signifies that almost every molecule could be precisely transferred, a fact further highlighted by not losing colocalization of donor and acceptor fluorophores before and after the transfer (Figure 4b). The absence of mobility in the red spots further corroborated the effectiveness of the dual cholesterol modification in achieving targeted and stable cargo delivery (Supporting Information Movie 2).

This modification signifies an advancement in the precision of cargo transport mechanisms via DNA origami structures, highlighting the potential for highly specific molecular and stoichiometric delivery systems in nanoscale applications. These findings highlight the importance of structural modifications in enhancing the specificity and efficacy of DNA origami‐based delivery systems.

## Conclusion

Understanding the interactions between vesicles and probes is not just of biophysical interest—it is a pathway to improving applications in medicine, diagnostics, and molecular biology. Built on the principles of affinity interactions, in this research, we have successfully developed a biosensor predicated on the flexibility and programmability of DNA origami structures. The vesicle sensor utilizes smFRET for the precise detection of lipid vesicles. Central to the effectiveness of the sensor is the hydrophobic ATTO647N dye‐modified DNA leash, whose conformational shifts in the presence or absence of lipid vesicles facilitate distinguishable FRET signals. This observed versatility in response is further augmented by the strategic positioning of cholesterol anchors, underscoring the pivotal role of precise molecular design in dictating vesicle‐sensor interactions. Moreover, the dynamic behavior of the sensor, as evidenced by the FRET shift upon vesicle interaction and the subsequent engagement of the hydrophobic probe with the vesicle, showcases its potential for targeted cargo transfer delivering a precisely adjustable number of molecules. Our findings on the different affinities of hydrophobic and hydrophilic dyes to lipid vesicles further amplify the importance of selecting appropriate probes for specific biosensing applications. Furthermore, the combination of the vesicle sensor with a strand displacement system highlighted the potential of the DNA origami nanosensing structures for conditional molecular transfer with initial vesicle sensing and subsequent cargo transfer. Our design introduces a new dimension to conditional cargo transfer. Unlike traditional delivery systems based on molecular logic gates (e.g., an AND gate that requires the presence of two chemical receptors), our system first senses the vesicle and then triggers cargo transfer through toehold‐mediated strand displacement.^[32]^ The final experiment with dual cholesterol‐labeled DNA strands highlights a significant improvement in the specificity of cargo transfer within DNA origami systems. Notably, this approach enabled to deliver exactly one desired cargo molecule per trapped vesicle, as opposed to the cargo spontaneously translocating to neighboring non‐trapped vesicles. This platform can therefore become the basis for loading lipid vesicles with a precisely defined number and combination of cargo molecules.

## Supporting Information

The authors have cited additional references within the Supporting Information.[^56^, ^57^, ^58^, ^59^, ^60^, ^61^, ^62^, ^63^, ^64^]

## Conflict of Interests

The authors declare no conflict of interest.

1

## Supporting information

As a service to our authors and readers, this journal provides supporting information supplied by the authors. Such materials are peer reviewed and may be re‐organized for online delivery, but are not copy‐edited or typeset. Technical support issues arising from supporting information (other than missing files) should be addressed to the authors.

Supporting Information

Supporting Information

Supporting Information

## Data Availability

The data that support the findings of this study are available from the corresponding author upon reasonable request.
